# Treatment decision for recurrences in non-small cell lung cancer during or after adjuvant osimertinib: an international Delphi consensus report

**DOI:** 10.3389/fonc.2023.1330468

**Published:** 2024-01-23

**Authors:** Myriam Mirza, Aseem Shrivastava, Cecile Matthews, Natasha Leighl, Calvin S. H. Ng, David Planchard, Sanjay Popat, Julia Rotow, Egbert F. Smit, Ross Soo, Masahiro Tsuboi, Fan Yang, Brendon Stiles, Christian Grohe, Yi-Long Wu

**Affiliations:** ^1^ Charles River Associates, Munich, Germany; ^2^ Charles River Associates, Cambridge, United Kingdom; ^3^ Department of Medical Oncology & Hematology, Princess Margaret Cancer Centre, Toronto, ON, Canada; ^4^ Department of Surgery, The Chinese University of Hong Kong, Hong Kong, Hong Kong SAR, China; ^5^ Department of Medical Oncology, Thoracic Group and International Center for Thoracic Cancers (CICT), Gustave Roussy, Villejuif, France; ^6^ Faculty of Medicine, Paris-Saclay University, Paris, France; ^7^ National Heart and Lung Institute, Imperial College, London, United Kingdom; ^8^ Lung Unit, The Royal Marsden, London, United Kingdom; ^9^ Division of Clinical Studies, The Institute of Cancer Research, London, United Kingdom; ^10^ Lowe Center for Thoracic Oncology, Dana-Farber Cancer Institute, Boston, MA, United States; ^11^ Department of Thoracic Oncology, Netherlands Cancer Institute, Amsterdam, Netherlands; ^12^ Department of Haematology-Oncology, National University Hospital, Singapore, Singapore; ^13^ Department of Thoracic Surgery and Oncology, National Cancer Center Hospital East, Kashiwa, Japan; ^14^ Thoracic Surgery Department, Peking University People’s Hospital, Beijing, China; ^15^ Thoracic Surgery and Surgical Oncology, Albert Einstein College of Medicine and Montefiore Medical Centre, New York, NY, United States; ^16^ Department of Pneumology, Evangelische Lungenklinik (ELK) Berlin, Berlin, Germany; ^17^ Guangdong Lung Cancer Institute, Guangdong Provincial People’s Hospital and Guangdong Academy of Medical Sciences, Guangzhou, China

**Keywords:** osimertinib, non-small cell lung cancer, adjuvant treatment, EGFR mutation, recurrence, treatment sequencing

## Abstract

**Introduction:**

Osimertinib is recommended by major guidelines for use in the adjuvant setting in patients with EGFR mutation-positive NSCLC following the significant improvement in disease-free survival observed in the Phase III ADAURA trials. Due to limited real-world data in the adjuvant setting, little guidance exists on how to approach potential recurrences either during or after the completion of the treatment. This study aimed to reach a broad consensus on key treatment decision criteria in the events of recurrence.

**Methods:**

To reach a broad consensus, a modified Delphi panel study was conducted consisting of two rounds of surveys, followed by two consensus meetings and a final offline review of key statements. An international panel of experts in the field of NSCLC (n=12) was used to provide clinical insights regarding patient management at various stages of NSCLC disease including patient monitoring, diagnostics, and treatment approach for specific recurrence scenarios. This study tested recurrences occurring 1) within or outside the central nervous system (CNS), 2) during or after the adjuvant-osimertinib regimen in NSCLC disease which is 3) amenable or not amenable to local consolidative therapy.

**Results:**

Panellists agreed on various aspects of patient monitoring and diagnostics including the use of standard techniques (e.g., CT, MRI) and tumour biomarker assessment using tissue and liquid biopsies. Consensus was reached on 6 statements describing treatment considerations for the specific NSCLC recurrence scenarios. Panellists agreed on the value of osimertinib as a monotherapy or as part of the overall treatment strategy within the probed recurrence scenarios and acknowledged that more clinical evidence is required before precise recommendations for specific patient populations can be made.

**Discussion:**

This study provides a qualitative expert opinion framework for clinicians to consider within their treatment decision-making when faced with recurrence during or after adjuvant-osimertinib treatment.

## 1 Introduction

A significant number of NSCLC patients harbor *EGFR* driver mutations (*EGFRm* NSCLC) which activate *EGFR* tyrosine kinase to have a ligand-independent activity, resulting in tumorigenesis (1–3). In US plus Europe and Asia, *EGFRm* NSCLC patients account for ~10-15% and ~30-50% of all NSCLC cases, respectively (4). The two most common *EGFR* mutations are short in-frame deletions of exon 19 and a point mutation in exon 21 which result in the substitution of leucine by arginine at codon 858 (L858R), together, accounting for ∼85% of all *EGFR* mutations in NSCLC (4). *EGFR* tyrosine kinase inhibitors (*EGFR*-TKIs) have been shown to significantly improve disease-free survival (DFS) in patients with resected early-stage *EGFRm* NSCLC (5, 6). Osimertinib is a third-generation *EGFR*-TKI approved in many countries around the world for both *EGFR*-TKI sensitizing (exon 19 deletion & L858R point mutation in exon 21) and T790M resistance mutations in advanced stage NSCLC patients (7). In 2020, primary analysis of the pivotal phase 3 ADAURA trial demonstrated a substantial DFS benefit in patients with EGFR-mutated NSCLC who underwent complete tumor resection, with hazard ratios of 0.17 (99% CI 0.11 to 0.26; p < 0.001) for stage II to IIIA disease and 0.20 (99% CI 0.14 to 0.30; p < 0.001) for stage IB to IIIA disease compared to placebo. Due to the significant improvement in DFS, the independent data monitoring committee recommended reporting the trial results two years earlier than originally planned, allowing patients to continue in the trial (8). Updated data with an additional 2 years of follow-up continued to show a sustained DFS benefit (hazard ratios of 0.23 in stage II and IIIA disease and 0.27 in stage IB and IIIA disease, respectively), In addition, recurrences among all the patients with stage IB to IIIA disease were less frequent with osimertinib (93 patients [27%]) than with placebo (205 patients [60%]). Recurrences in the osimertinib group included distant metastases only (45 patients [13%]), local/regional only (42 patients [12%]), as well as both local/regional and distance (6 patients [2%]) (9).

Most recently, published data on overall survival (OS) in the overall population (patients with stage IB to IIIA disease) report an OS HR of 0.49 (95% CI 0.34 to 0.70; p < 0.0001) with a 5-year OS rate of 88% with osimertinib vs 78% with placebo. In stage II–IIIA disease, OS HR was reported to be 0.49 (95% CI 0.33 to 0.73; p=0.0001) and the 5-year OS rate was 85% with osimertinib vs 73% with placebo. The median OS was not reached in either population or treatment group (10).

While adjuvant-osimertinib demonstrated an unprecedented patient benefit in terms of OS improvements, there is a need to understand better the optimal management of patients who show tumor recurrence either during or after the completion of the adjuvant-osimertinib regimen. Given the anticipated emergence of a patient population with disease relapse following adjuvant osimertinib treatment, and the absence of real-world data or trial data, creating formal guidelines on how to approach and manage recurrent patients either during or after completion of adjuvant-osimertinib is not yet possible. The knowledge gap regarding the appropriate approach for patient monitoring, diagnostics, and treatment sequencing decisions in cases of various recurrence scenarios can be bridged via clinical consensus studies. Recent consensus studies echoed the need for further clinical trial data to create formal guidelines (11, 12) and outlined the appropriate treatment options (including osimertinib) for different recurrence scenarios (12). In this consensus paper, we discuss the key clinical factors that can be considered during treatment decision-making as well as clinical value of osimertinib for various recurrence scenarios.

## 2 Materials and methods

The study utilized a modified Delphi method which included two rounds of surveys, followed by two consensus meetings and a final offline review by an expert panel. The key topics addressed in this study are listed in **Table 1**.

**Table 1:** Key topics addressed in this study^i^.

**1**	**General treatment decision influencers within NSCLC and recurrence scenarios**: Patient characteristics and history including age, smoking status, the initial stage of the disease, and the subsequent treatment methodology
**2**	**Patient monitoring approach within adjuvant setting:** Different techniques used and the monitoring frequency both during and after the completion of the adjuvant-osimertinib regimen
**3**	**Diagnostic approach upon recurrence suspicion:** Different diagnostic approaches and their potential impact on the ongoing adjuvant-osimertinib regimen
**4**	**Treatment and management approaches for CNS and ex-CNS recurrence:** Treatment decision-making process considering recurrence type, timing, and other clinically relevant factors^ii^
**5**	**Variation in patient management based on the recurrence scenarios:** Potential differences in the patient monitoring, diagnostic and/or treatment approaches for the following distinct recurrence scenarios: i. Ex-CNS or CNS recurrences during the adjuvant-osimertinib regimen that are amenable to local consolidative therapy^iii^ ii. Ex-CNS or CNS recurrences post-adjuvant osimertinib regimen that are amenable to local consolidative therapy iii. Ex-CNS or CNS recurrences during the adjuvant-osimertinib regimen that are not amenable to local consolidative therapy iv. Ex-CNS or CNS recurrences post-adjuvant-osimertinib regimen that are not amenable to local consolidative therapy

i The table displays the key topics related to disease recurrence events during or after adjuvant osimertinib treatment for EGFRm NSCLC.

^ii^Clinical considerations of treatment sequencing decisions and potential geographic differences in the recommended treatment sequencing were also captured.

^iii^Local consolidative therapy includes surgery, ablation (percutaneous/endoscopic ablations including thermal/cryo ablation), radiotherapy, conformal radiotherapy, or stereotactic body radiation therapy (SBRT).

### 2.1 Panel selection

In this study, an international panel of experts was recruited with significant expertise in NSCLC as well as patient management with *EGFR*-TKIs or other systemic therapies (n=12). Experts who fulfil the following criteria were selected as panelists in this study: a physician specializing in NSCLC (medical oncologist or thoracic surgeon); based in a specialist lung cancer treatment and research center; significant years of experience in practice since completing residence/fellowship; over 60% of combined professional time dedicated to clinical practice and research activities related to NSCLC; regularly treating and managing patients across all stages of NSCLC (stage I-III); active advisor/member of a national or international society for lung cancer with participation in guideline creation for NSCLC in the last 5 years; has published on the topic of stage I-III NSCLC in international peer-reviewed journals within the last 5 years.

The steering committee consisted of two medical oncologists and one thoracic surgeon from different geographies (Asia, Europe, and the USA) to ensure geographic as well as different clinical expertise were incorporated in the development of materials.

### 2.2 Delphi methodology and statement development

Both surveys were composed of a series of open and close ended question and shared via email. All materials tested in the study were co-developed and reviewed by the steering committee. In Survey 1, panelists were presented with six hypothetical patient case studies representing distinct real-world *EFGRm* NSCLC patients who recur during or after the treatment with adjuvant-osimertinib and were surveyed on their approach to patient monitoring, diagnostic workup, and treatment sequencing (see **Supplementary Data 1** – **Table 1** for hypothetical patient cases). Most questions were asked in an open-ended style to capture individual approach as well as clinical considerations. After analysis, topics that reached clinical consensus were reported back in Survey 2 as anonymized consolidated feedback and those that did not reach consensus were further probed using new clinical statements based on insights from Survey 1 (see **Supplementary Data 2** for both survey 1 and 2). The insights gathered from Survey 2 were analyzed to find topics of clinical consensus. Statements or insights where clinical consensus was not achieved were brought forward to a series of consensus meetings (see **Supplementary Data 1** – **Table 2**).

**Table 2:** Survey 1 and Survey 2 outcomes – Major insights with an overall panellists’ agreement.

Survey 1 and 2 Outcomes			
Key topic	Insights		Agreement reached
**Patient monitoring during or after adjuvant-osimertinib regimen**	1	Patients monitoring during or after ADAURA regimen is conducted using CT scan and brain MRI in line with local guidelines.	Survey 1
	2	Additional patient monitoring techniques like MRD and blood tests (e.g., Carcinoembryonic antigen assay) can be used as add-on techniques but caution is recommended as currently there is no clinically validated MRD assay	Survey 2
	3	Tissue biopsy is performed for histological and molecular analysis to look for actionable targets (e.g., EGFR, PDL-1, others) in all feasible cases	Survey 1
	4	Liquid biopsies are performed when tissue biopsy is difficult or as a second diagnostic method. Factors such as patient or doctor preference for non-invasive procedure also influence the decision to perform liquid biopsies but they have secondary priority	Survey 2
	5	NGS or other molecular analysis procedures are always performed if progression is observed	Survey 2
	6	In case of recurrence, osimertinib is used during the entire diagnostic workup and up until a new treatment regimen is decided • Use of osimertinib can help avoid disease flare (as observed in metastatic setting) and decrease the risk of progression in the CNS • Osimertinib might be part of the new treatment regimen depending on the re-biopsy results	Survey 2
**Treatment approach: ex-CNS and/or CNS recurrence (amenable to local consolidative therapy)**	7	Treatment for oligometastatic distant recurrence includes local consolidative therapies – surgery, percutaneous/endoscopic ablations including thermal/cryo ablation, radiotherapy, conformal radiotherapy, or stereotactic body radiation therapy (SBRT)	Survey 1
	8	Treatment strategies for CNS recurrence include combination of local consolidative therapies (surgery or radiotherapy) with systemic therapies • Surgery will be performed only when anatomically feasible • Stereotactic radiosurgery (SRS) and whole-brain radiation therapy (WBRT) are the preferred radiotherapy options for oligometastatic and disseminated CNS recurrences, respectively • Continuation of osimertinib is a treatment option depending on the recurrence histology	Survey 1
	9	In case of recurrence during the adjuvant osimertinib regimen that is amenable to local consolidative therapy, local consolidative therapy is performed in parallel to continuation of adjuvant osimertinib use, if possible	Survey 2
	10	In case of recurrence after the adjuvant osimertinib regimen is completed, local consolidative therapy is performed in parallel to rechallenge with osimertinib, if possible	Survey 2
**Treatment approach: ex-CNS and/or CNS recurrence (not amenable to local consolidative therapy)**	11	Treatment approach for disseminated distant recurrence involves systemic therapies based on the newly obtained histology. Systemic therapies mentioned include targeted drugs against the resistance mechanism (other TKIs), chemotherapy, immunotherapy, and combinational approach (e.g., chemo + TKI, chemo + immunotherapy)^i^	Survey 1
	12	In case of ex-CNS or CNS recurrence during the adjuvant osimertinib regimen and local consolidative therapy is not an option, systemic therapy based on the re-biopsy result is recommended with potential to include osimertinib depending on patient case	Survey 2
	13	In case of recurrence ex-CNS and/or CNS after completion of adjuvant osimertinib regimen, therapeutic approach will be based on re-biopsy results and in conjunction with the MDT with potential to include osimertinib depending on patient case	Survey 2

i Combination treatment approach with osimertinib and immunotherapy was flagged as unsuitable by KEEs and additional caution was recommended in case of sequential use.

Two virtual consensus meetings were held where a final set of statements were discussed and amended live during the meeting. The level of consensus on the amended statements was probed through anonymous polls. The statement modification process was iterated until an overall clinical consensus (≥80% panelist agreement) was achieved for all six statements (see **Supplementary Data 1** – **Table 3** for the evolution of the survey statements pre- and post-consensus meeting). The final set of consensus statements were shared with all panelists for offline review and to capture their final level of agreement.

**Table 3:** Final consensus statements with an overall panelist agreement.

Recurrence scenarios	Final Consensus Statement		Panelist Agreement
**ex-CNS or CNS recurrence amenable to local consolidative therapy**	1	If a patient experiences an ex-CNS or CNS recurrence amenable to local consolidative therapy during the adjuvant-osimertinib regimen, in absence of existing evidence, I would use the adjuvant-osimertinib regimen in parallel with local consolidative therapy, considering the risk of toxicities, site of recurrence, method of local consolidative therapy and after discussion with the MDT	**Consensus reached** 11 out of 12 panelists agreed
	2	If a patient experiences an ex-CNS or CNS recurrence amenable to local consolidative therapy after completion of the adjuvant-osimertinib regimen, I would consider rechallenging with osimertinib as an option after local consolidative therapy, after discussion with MDT	**Consensus reached** 10 out of 12 panelists agreed
**ex-CNS and/or CNS recurrence during adjuvant-osimertinib regimen not amenable to local consolidative therapy**	3	If a patient experiences CNS-only recurrence not amenable to local consolidative therapy during the adjuvant-osimertinib regimen, I would consider continuing with osimertinib as a part of the overall treatment strategy when a patient has asymptomatic progression, if fits with the best clinical practice	**Consensus reached** 10 out of 12 panelists agreed
	4	In the absence of randomized evidence, if a patient experiences ex-CNS recurrence not amenable to local consolidative therapy during the adjuvant-osimertinib regimen, I would consider continuation with osimertinib as a part of the overall treatment strategy if a patient has asymptomatic progression and if fits with the current guideline recommendations	**Consensus reached** 10 out of 12 panelists agreed
**ex-CNS and/or CNS recurrence post-adjuvant-osimertinib regimen not amenable to local consolidative therapy**	5	If a patient experiences an ex-CNS and/or CNS recurrence after completion of the adjuvant-osimertinib regimen, and local consolidative therapy is not an option, I would rechallenge with monotherapy osimertinib or osimertinib as a part of the overall treatment strategy irrespective of when the recurrence happens after treatment completion, if fits with the best clinical practice and is supported by re-biopsy in all feasible cases	**Consensus reached** 12 out of 12 panelists agreed
**All ex-CNS and/or CNS recurrence types**	6	If a patient experiences an ex-CNS and/or CNS recurrence during or after the completion of the adjuvant-osimertinib regimen and I choose to continue with osimertinib as part of the overall treatment strategy, I would continue the osimertinib therapy until the clinical situation mandates a change or stop in therapy	**Consensus reached** 11 out of 12 panelists agreed

### 2.3 Defining consensus

Closed statements were ranked on the Likert scale of 1 to 9, where 1 equals “strongly disagree with the statement,” and 9 equals “strongly agree with the statement,”. Likert scale rating of 7 or higher for a given statement from a minimum of 80% of panelists was defined as a threshold for a consensus on the statement. In contrast, a rating of 3 or lower for a given statement from a minimum of 80% of panelists indicated consensus had been reached of disagreement with the statement.

### 2.4 Limiting bias

To limit bias, all surveys were conducted anonymously, and identity of experts was revealed only during the consensus meetings. Furthermore, results from rating exercises were provided as median scores and anonymous votes were held to finalize the consensus statements. In addition, offline review of the final statements was conducted individually without revealing the level of agreement from other panelists.

An independent third-party vendor, Charles River Associates (CRA) designed the survey, moderated the consensus meetings, analyzed the data and supported the manuscript development. The sponsor of the study (AstraZeneca Ltd.) did not participate in consensus meetings.

## 3 Results

Surveys 1 and 2 were used to gather a greater understanding of the factors influencing treatment sequencing within the adjuvant-osimertinib setting, including recurrence type and timing. A summary of the key insights gathered from Surveys 1 and 2 is shown in **Table 2**.

### 3.1 Survey 1

In Survey 1, six distinct hypothetical NSCLC patient case studies were used to understand how panelists approach management of different patient types and understand the potential differences under varying recurrence scenarios. Patients within the case studies had varying *EGFRm* mutations, ethnicities (Asian/non-Asian), age groups (40-65 years old), smoking status, past experience with adjuvant chemotherapies, recurrence either during or after adjuvant-osimertinib and details of the recurrence. All patients within the case studies were given a performance status (PS) score of 1.

Overall, panelists agreed that all the hypothetical patient cases are representative of real-world profiles and the use of adjuvant-osimertinib in these hypothetical patient cases is approved under the ADAURA label. Panelists also agreed on the monitoring and diagnostic approaches laid out, albeit frequency of monitoring and how molecular analysis is conducted remained unclear. Moreover, although some panelists mentioned the use of monitoring techniques such as minimal residual disease (MRD) tests and blood tests (e.g., CEA - carcinoembryonic antigen), it was unclear if these techniques would be used for all patients or in specific circumstances. Finally, there was a lack of agreement on the value of liquid biopsy within the diagnostic process. Overall, despite no diagnostic procedural differences between oligometastatic and disseminated recurrences being reported there remained a lack of clarity as to whether adjuvant-osimertinib regimen should be continued during diagnostic workup and up until a new treatment strategy is confirmed.

Panelists agreed on treatment approaches for local and distant oligometastatic CNS/ex-CNS recurrence as well as disseminated CNS/ex-CNS recurrence. However, treatment sequencing decision for both ex-CNS and CNS recurrences and the associated driving factors were unclear. No consensus was observed on how patients treated with the preferred initial treatment (e.g., ablative therapy) are therapeutically followed up and to what extent osimertinib would be considered a therapeutic option.

### 3.2 Survey 2

Gaps identified in Survey 1 were explored in Survey 2, where agreement was reached on all outstanding aspects of diagnosis and monitoring. Panelists were presented with different scenarios to better understand the treatment sequencing drivers and how adjuvant-osimertinib would be considered in the overall treatment strategy. These scenarios focused on understanding the impact of recurrence location (CNS versus ex-CNS), amenability to local consolidative therapy, timing (during or after adjuvant-osimertinib regimen) as well as time points (3, 6, 18 months after initiation of adjuvant-osimertinib versus 3, 12 and 36 months after completion with adjuvant-osimertinib regimen).

While panelists agreed that continuing with adjuvant-osimertinib is clinically suitable during the diagnostic process and can be considered within treatment decision-making in the described CNS/ex-CNS recurrence scenarios, how its use would be decided remained unclear. Furthermore, there was no consensus on how long treatment with osimertinib would continue within recurrence scenarios and what considerations would influence this decision. Finally, some panelists suggested temporarily pausing the adjuvant-osimertinib regimen upon recurrence and resuming after ablative therapy, but the recommended pause duration and its applicability to all ablative therapy procedures are not clear. For recurrences after completing the adjuvant-osimertinib regimen, a combination of ablative therapy and osimertinib rechallenge was proposed, but it remains unclear whether the rechallenge would be performed alongside ablative therapy or after its completion.

### 3.3 Consensus meeting

Gaps in key treatment decision factors and the value of osimertinib across tested recurrence scenarios were addressed during 2 virtual consensus meetings, where experts amended wording of original proposed statements in line with best clinical practice and experience given limited randomized evidence. A summary of the statements that reached a consensus is shown in **Table 3**.

## 4 Discussion

A lack of clinical guidance on how to treat recurrences during or after the completion of the adjuvant-osimertinib regimen has been raised in recent publications (11, 12). Our Delphi consensus study surveyed an international panel of experts on patient-specific as well as recurrence scenario-specific considerations when making treatment decisions in the absence of extensive clinical data and formalized guidelines.

Our study approach enables experts to share their own opinions based on clinical experience, and, where relevant, knowledge of the ADAURA phase 3 data, with the aim of fostering the integration of these viewpoints. Overall, our research shows a high consensus regarding patient considerations and approaches to various recurrence scenarios. Statements and considerations that garnered consensus can serve as valuable guidance in clinical practice when combined with a tailored patient approach and recommendations from multidisciplinary teams.

### 4.1 Key considerations for adjuvant-osimertinib and *EGFRm* NSCLC patient monitoring & diagnostic work-up

Our results show that the decision to use adjuvant-osimertinib is not driven by patient’s gender, ethnicity, age, smoking or alcohol use status, or previous treatment experience with adjuvant-chemotherapy. However, the decision to use adjuvant-osimertinib might change if a patient has poor PS score (PS >1), has high comorbidities or is likely to be less compliant with the dosing regimen.

Furthermore, patients receiving adjuvant-osimertinib should be monitored using local guidelines which broadly align with international guidelines such as ESMO (**Table 2** – statement 1).

In our study, tissue biopsy is recommended in all feasible cases in case of progression to assess the suitability of different treatment options based on tumor biomarker/mutation profile. The diagnostic result from liquid biopsy was highlighted to be less sensitive and contingent on site and burden of the recurrence (**Table 2** – statements 3-5). Therefore, only when tissue biopsy is not possible should liquid biopsy be used as it is unable to capture histologic transformation and has limited sensitivity from amplifications and fusions (12). Moreover, liquid biopsies may give false negative or positive results, especially in cases of low volume disease with lower than threshold circulating ctDNA (13). These recommendations are echoed in the ESMO expert consensus on the management of *EGFRm* NSCLC (12). The panel’s consensus is to continue use of adjuvant-osimertinib during the diagnostic workup and up until a new treatment regimen is decided for patients who present with recurrence during the adjuvant-osimertinib regimen. The continued use of osimertinib can help avoid disease flare, a well-established scenario for metastatic disease, and to additionally protect against CNS progression (**Table 2** – statement 6) (9, 14, 15).

### 4.2 Key considerations in treatment sequencing for adjuvant-osimertinib recurrence scenarios

Across all tested scenarios, treatment decisions are taken on a patient-by-patient basis and taking input from the multi-disciplinary team (MDT), aligning with major guidelines (16–21). The following key clinical criteria should be considered during the treatment sequencing decision process (**Table 2** – statements 7-13):

- Pattern and location – Number of lesions, size and location determine if the recurrence is oligometastatic and amenable to local consolidative therapy or disseminated and requires treatment with a systemic therapy. Furthermore, limited metastases which may include lesions in contralateral lung, lymph node, CNS or other organs could be managed with a combination of local consolidative therapy and a systemic therapy. Panelists did not rule out the treatment value of osimertinib in various recurrence scenarios solely based on pattern and location.
- Timing of recurrence – whether and when recurrence occurs during or after the adjuvant-osimertinib regimen can indicate if the recurrent lesion is sensitive or resistant to osimertinib in addition to genetic profiling. Recurrence during the adjuvant-osimertinib regimen (especially ex-CNS) might be a sign of acquired resistance to osimertinib, while recurrence after the completion of the regimen might indicate a disease flare post-treatment cessation.
- Molecular characterization of the recurrence – Confirming molecular histology or biomarker profile informs the presence of any actionable target and is necessary when considering further treatment with osimertinib.

It is important to note here that while panelists acknowledge osimertinib to be a valuable treatment option for patients with complex clinical presentations, including the emergence of distinct resistance mechanisms (e.g., MET amplification, PIK3CA mutation, BRAF mutation) and/or progression to acquire other genetic alterations (e.g., ALK mutation), these specific recurrence scenarios were not explored in this study.

#### 4.2.1 Therapeutic value of osimertinib: ex-CNS or CNS recurrences during the adjuvant-osimertinib regimen that are amenable to local consolidative therapy

For ex-CNS or CNS recurrences which occur during the adjuvant-osimertinib regimen and are amenable to local consolidative therapy (surgery, ablation, or radiotherapy), the panel recommended to continue adjuvant-osimertinib treatment along with local consolidative therapy if found clinically suitable (**Table 3** – consensus statement 1). A consensus was achieved in pausing osimertinib regimen during radiotherapy due to severe toxicity risk in case of combined use, however, length of pause was not specified. Pausing the use of therapies such as osimertinib during radiotherapy was also recently highlighted in published consensus recommendations by the EORTC-ESTRO OligoCare consortium, where a consensus was reached to not perform SBRT within one week of the administration of anti-EGFR antibody (22).

However, in the absence of safety data in combining local consolidative therapy with osimertinib treatment, recommendations on pausing or continuing with osimertinib during ablative or surgical procedures could not be reached and instead in the absence of treatment guidelines, the decision to pause osimertinib should be based on treatment experience from the metastatic setting, i.e., considering the extent of recurrence including location, size and number of the lesions (note that the treatment guidelines for metastatic settings are also not yet established). Additionally, caution was recommended when patients are given other drugs (e.g., prophylactic antibiotics before local consolidative therapy) that could show drug-drug interactions with osimertinib.

#### 4.2.2 Therapeutic value of osimertinib: ex-CNS or CNS recurrences post-adjuvant osimertinib regimen that are amenable or not amendable to local consolidative therapy

While the place of osimertinib within the treatment strategy is dependent on multiple factors (see Section 4.2), the treatment value of rechallenging with osimertinib was thought to increase as duration between time to recurrence and completion of the adjuvant-osimertinib regimen increases. However, no consensus was achieved on the minimum recurrence free time (3, 12 or 36 months) to consider osimertinib for rechallenge.

In cases of post-adjuvant osimertinib regimen recurrence scenarios that are amenable to local consolidative therapy and rechallenging with osimertinib is suitable, the consensus is to rechallenge with osimertinib after the completion of the local consolidative therapy (**Table 3** – consensus statement 2), especially in the case of radiotherapy where parallel use of osimertinib is not recommended due to the risk of toxicities. However, it is still to be determined whether stand-alone local consolidative therapy is sufficient and has curative potential in some patient cases or whether it should always be followed with osimertinib rechallenge to prevent potential distant metastasis.

In case of post-adjuvant osimertinib regimen recurrence scenarios that are not amenable to local consolidative therapy, rechallenge with osimertinib may be of high clinical value and should therefore be considered either as osimertinib monotherapy or as a part of treatment strategy involving other therapies (**Table 3** – consensus statement 5).

#### 4.2.3 Therapeutic value of osimertinib: ex-CNS or CNS recurrences during the adjuvant-osimertinib regimen that are not amenable to local consolidative therapy

Our study found that ex-CNS recurrence in the first 6 months is an indication of treatment failure of adjuvant-osimertinib, requiring change of treatment to either chemotherapy, other targeted therapies, or a combination of both. However, for later recurrences, continuation of osimertinib as a part of a broad treatment strategy could be potentially valuable (**Table 3** – consensus statement 4) as it may prevent brain metastases, especially in case of ex-CNS recurrence.

For CNS-only recurrences, the consensus is that continuation of osimertinib as a part of a broad treatment strategy could be an effective treatment option (**Table 3** – consensus statement 3). For indolent CNS recurrences, continuation of osimertinib monotherapy could also be a valuable treatment option, given the CNS recurrence could stem from underexposure to osimertinib. However, further evidence is needed on the appropriate osimertinib dosing regimen and/or combination with other therapies before these treatment approaches are considered outside clinical trials. The use of osimertinib for both ex-CNS and CNS-only recurrence was identified to be more suitable for patients with asymptomatic progression which indicates that the tumor growth is gradual and the risk of resistance to osimertinib is relatively lower than within symptomatic progression (**Table 3** – consensus statements 3 & 4).

#### 4.2.4 Osimertinib treatment duration for all indicated CNS and/or ex-CNS recurrences

In cases where osimertinib was considered as a treatment option within the recurrence scenarios, the consensus was that treatment would continue until disease progression or toxicities were observed, or patient quality of life deteriorated. For a low progression risk patient (e.g., indolent oligometastatic progression), the duration of osimertinib treatment should be limited and a decision to stop treatment should be taken after monitoring the efficacy and patient’s health profile and considering the safety and tolerance profile of osimertinib and patient wishes (**Table 3** – consensus statement 6). For a high progression risk patient, the treatment duration would be longer compared to a low progression risk patient with an aim to avoid any residual disease flare after osimertinib treatment cessation.

## 5 Conclusion

Outcomes from the phase 3 ADAURA trial show significant improvements in DFS and OS for EGFR-mutated NSCLC patients with stage IB to IIIA disease. Nonetheless, patterns of recurrence were reported within the osimertinib group, although lower compared with placebo (9, 10). In the absence of clinical guidance on how to treat recurrences during or after the completion of the adjuvant-osimertinib regimen, our consensus study offers a qualitative framework for clinicians in such scenarios, drawing from international expert consensus. While recognizing the importance of additional clinical data from trials and real-world settings, the study provides broader treatment considerations. It also considers osimertinib’s efficacy, as supported by FLAURA, AURA3, and ADAURA trials (7, 9, 23), with panelists acknowledging its potential benefits across various recurrence scenarios, including oligometastatic CNS recurrences and CNS metastases. In addition, panelists acknowledged the potential treatment value of combination therapies using osimertinib with local consolidative therapy, chemotherapy, and other systemic therapies; however, further efficacy and safety data is needed. The suitability of each combination approach under different recurrence scenarios was not tested in this study indicating the need for more discussion on clinical experience in the absence of concrete data.

## Data availability statement

The original contributions presented in the study are included in the article/**Supplementary Material**. Further inquiries can be directed to the corresponding author.

## Author contributions

MM: Conceptualization, Investigation, Methodology, Project administration, Supervision, Writing – original draft, Writing – review & editing. AS: Conceptualization, Formal Analysis, Investigation, Methodology, Project administration, Writing – original draft, Writing – review & editing. CM: Conceptualization, Funding acquisition, Methodology, Project administration, Supervision, Writing – review & editing. NL: Writing – review & editing. CN: Writing – review & editing. DP: Writing – review & editing. SP: Writing – review & editing. JR: Writing – review & editing. ES: Writing – review & editing. RS: Writing – review & editing. MT: Writing – review & editing. FY: Writing – review & editing. BS: Supervision, Writing – review & editing. CG: Supervision, Writing – review & editing. Y-LW: Supervision, Writing – review & editing.
