# Supplementary material for: Treatment decision for recurrences in non-small cell lung cancer during or after adjuvant osimertinib: an international Delphi consensus report
Source: Front Oncol. 2024 Jan 23;13:1330468. doi: 10.3389/fonc.2023.1330468 (PMC10845045; doi:10.3389/fonc.2023.1330468)
Supplement: Supplementary file 1 [file DataSheet_1.docx]

# Table1 – Hypothetical Patient Case Development

1:1 meetings with the steering committee members were conducted to develop six hypothetical patient cases representative of the real-world EGFRm NSCLC patients who recur during or after the treatment with adjuvant-osimertinib. These patient cases encompassed different recurrence scenarios with regards to patient characteristics, recurrence timing, location, pattern and formed the basis of the Survey 1.

| Criteria | Case Study | | | | | |
| --- | --- | --- | --- | --- | --- | --- |
|  | **1** | | **2** | | **3** | |
| Patient characteristics | Non-Asian, Male | | Non-Asian, Female | | Asian, Male | |
|  | 40 – 60 years | | >70 years | | 60 – 70 years | |
|  | PS ≤ 1 | | PS ≤ 1 | | PS ≤ 1 | |
| Experience with Adjuvant Chemotherapy | Yes | | Yes | | No | |
| Recurrence during or after the ADAURA regimen | During | | After | | **a)** During | **b)** After |
| Details of recurrence | **a)** Distant recurrence, oligo-metastasis | **b)** Distant disseminated recurrence | **a)** Distant recurrence, oligo-metastasis | **b)** Distant disseminated recurrence | Local recurrence, no signs of distant metastasis | |

| Criteria | Case Study | | | | | |
| --- | --- | --- | --- | --- | --- | --- |
|  | **4** | | **5** | | **6** | |
| Patient characteristics | Non-Asian, Male | | Non-Asian, Female | | Asian, Female | |
|  | >70 years | | 40 – 60 years | | 60 – 70 years | |
|  | PS ≤ 1 | | PS ≤ 1 | | PS ≤ 1 | |
| Experience with Adjuvant Chemotherapy | Yes | | Yes | | No | |
| Recurrence during or after the ADAURA regimen | During | | After | | **a)** During | **b)** After |
| Details of recurrence | **a)** CNS only, oligo-metastasis | **b)** CNS only, disseminated recurrence | **a)** CNS only, oligo-metastasis | **b)** CNS only, disseminated recurrence | Distant recurrence and CNS | |

# Table 2 - Summary of consensus statements and the associated median rating on the Likert scale per key topic

| **Key topic** | **Statement** | | **Median rating on the Likert scale** |
| --- | --- | --- | --- |
| **Patient monitoring & diagnostic workup** | 1 | In addition to monitoring patients in adjuvant-osimertinib through CT/PET CT scan and Brain MRI, I would conduct Minimal residual disease (MRD) test | 3.5 |
|  | 2 | In addition to monitoring patients in adjuvant-osimertinib through CT/PET CT scan and Brain MRI, I would conduct blood tests | 4 |
|  | 3 | During the diagnostic work-up, I perform liquid biopsies when:  a. Tissue biopsy is difficult | 9 |
|  |  | b. Non-invasive procedure is preferred by the patient | 8 |
|  |  | c. Non-invasive procedure is preferred by the doctor | 7 |
|  |  | d. I do not perform liquid biopsies | 1 |
|  | 4 | I would always perform NGS or other molecular analysis procedures to understand the histology of the recurrence | 9 |
|  | 5 | I would continue the prescribed osimertinib regimen during the entire diagnostic work-up for confirming the recurrence/type of recurrence | 8 |
|  | 6 | Upon confirmation of the recurrence/type of recurrence, I would continue the prescribed osimertinib regimen until a treatment option can be implemented | 8.5 |
| **Treatment approach for ex-CNS recurrence** | **If 6 months after the start of the adjuvant-osimertinib regimen, a distant recurrence presented ex-CNS and lesions were deemed amenable for ablative therapy by the multidisciplinary team (MDT)** | | |
|  | 7 | a. I would use ablative therapy and continue treatment with adjuvant-osimertinib | 8.5 |
|  |  | b. I would use systemic chemotherapy or other systemic non-TKI options (e.g., immune-oncology therapy) either alone or in combination | 1 |
|  | 8 | I would use ablative therapy and continue treatment with adjuvant-osimertinib if the same described recurrence occurred 3 months after the start of the adjuvant-osimertinib regimen | 8.5 |
|  | 9 | I would use ablative therapy and continue treatment with adjuvant-osimertinib if the same described recurrence occurred 1.5 years after the start of the adjuvant-osimertinib regimen | 8.5 |
|  | 10 | I would use ablative therapy and continue treatment with adjuvant-osimertinib if the same described recurrence occurred 3 months after the completion of the adjuvant-osimertinib regimen | 8 |
|  | 11 | I would use ablative therapy and continue treatment with adjuvant-osimertinib if the same described recurrence occurred 1 year after the completion of the adjuvant-osimertinib regimen | 8 |
|  | 12 | I would use ablative therapy and continue treatment with adjuvant-osimertinib if the same described recurrence occurred 3 years after the completion of the adjuvant-osimertinib regimen | 9 |
|  | **6 months after the start of the adjuvant-osimertinib regimen, a distant recurrence presents ex-CNS which is deemed not amenable for ablative therapy by the multidisciplinary team (MDT). Instead, the MDT agree to treat the patient with systemic chemotherapy considering the re-biopsy results show no targetable resistance mutation** | | |
|  | 13 | a. I agree with the MDTs approach | 9 |
|  |  | b. I would continue treatment with adjuvant-osimertinib in parallel to the treatment approach | 3.5 |
|  |  | c. I would continue treatment with adjuvant-osimertinib following the treatment approach | 1 |
|  | 14 | I would use adjuvant-osimertinib the same way if the same described recurrence occurred 3 months from the start of the adjuvant-osimertinib regimen | 8.5 |
|  | 15 | I would use adjuvant-osimertinib the same way if the same described recurrence occurred 1.5 years from the start of the adjuvant-osimertinib regimen | 8 |
|  | 16 | a. I would change the therapy approach if the same described recurrence occurred 3 months after the completion of the adjuvant-osimertinib regimen | 2 |
|  |  | b. I would rechallenge with osimertinib in this scenario | 8 |
|  | 17 | a. I would change the therapy approach if the same described recurrence occurred 3 months after the completion of the adjuvant-osimertinib regimen | 2 |
|  |  | b. I would rechallenge with osimertinib in this scenario | 9 |
|  | 18 | a. I would change the therapy approach if the same described recurrence occurred 3 years after the completion of the adjuvant-osimertinib regimen | 2 |
|  |  | b. I would rechallenge with osimertinib in this scenario | 9 |
| **Treatment approach for CNS recurrence** | **If 6 months after the start of the adjuvant-osimertinib regimen, a distant non-symptomatic recurrence presented in the brain (PS = 1) and lesions were deemed amenable for ablative therapy by the multidisciplinary team (MDT)** | | |
|  | 19 | a. I would use ablative therapy (including surgery, radiotherapy, radiofrequency or others) that is most suitable for the patient and continue treatment with adjuvant-osimertinib | 9 |
|  |  | b. I would use systemic chemotherapy or other systemic non-TKI options (e.g., immune-oncology therapy) either alone or in combination | 1 |
|  | 20 | I would use ablative therapy and continue Tx with adjuvant-osimertinib if the same described recurrence occurred 3 months after the start of the adjuvant-osimertinib regimen | 8.5 |
|  | 21 | I would use ablative therapy and continue Tx with adjuvant-osimertinib if the same described recurrence occurred 1.5 years after the start of the adjuvant-osimertinib regimen | 9 |
|  | 22 | a. I would change the therapy approach if the same described recurrence occurred 3 months after the completion of the adjuvant-osimertinib regimen | 1.5 |
|  |  | b. I would rechallenge with osimertinib in this scenario | 8.5 |
|  | 23 | a. I would change the therapy approach if the same described recurrence occurred 1 year after the completion of the adjuvant-osimertinib regimen | 1.5 |
|  |  | b. I would rechallenge with osimertinib in this scenario | 8.5 |
|  | 24 | a. I would change the therapy approach if the same described recurrence occurred 3 years after the completion of the adjuvant-osimertinib regimen | 1 |
|  |  | b. I would rechallenge with osimertinib in this scenario | 9 |
|  | **25** | a. I would use ablative therapy (including surgery, radiotherapy, radiofrequency or others) that is most suitable for the patient and continue treatment with adjuvant-osimertinib if a distant symptomatic recurrence presented in the brain (PS = 1) and lesions were deemed amenable for ablative therapy by the multidisciplinary team (MDT) | 8 |
|  |  | b. I would use the same treatment approach regardless of when recurrence occurs | 7.5 |
|  | **26** | a. I would change the therapy approach if a distant non-symptomatic recurrence presented in the brain and lesions were deemed not amenable for ablative therapy | 5 |
|  |  | b. I would continue with adjuvant-osimertinib in this scenario | 8.5 |
|  |  | c. I would use the same treatment approach regardless of when recurrence occurs | 7.5 |
|  | **27** | a. I would change the therapy approach if a distant symptomatic recurrence presented in the brain and lesions were deemed not amenable for ablative therapy | 8 |
|  |  | b. I would continue with adjuvant-osimertinib in this scenario | 3 |
|  |  | c. I would use the same treatment approach regardless of when recurrence occurs | 7 |
|  | **28** | a. I would change the therapy approach if a distant non-symptomatic recurrence presented in both the brain and ex-CNS and lesions were deemed not amenable for ablative therapy | 6 |
|  |  | b. I would continue with adjuvant-osimertinib in this scenario | 2.5 |
|  |  | c. I would use the same treatment approach regardless of when recurrence occurs | 7 |
|  | **29** | a. I would change the therapy approach if a distant symptomatic recurrence presented in both the brain and ex-CNS and lesions were deemed not amenable for ablative therapy | 6.5 |
|  |  | b. I would continue with adjuvant-osimertinib in this scenario | 2.5 |
|  |  | c. I would use the same treatment approach regardless of when recurrence occurs | 7 |

# Table 3 - Evolution of draft consensus statements into final consensus statements during the consensus meeting

| **Key topic** | **Pre-Consensus Meeting Statement** | **Post-Consensus Meeting Statement**  *(final consensus statements)* |
| --- | --- | --- |
| **ex-CNS or CNS recurrence** (amenable to local consolidative therapy) | **A.** If a patient experiences an ex-CNS or CNS recurrence amenable to ablative therapy during the adjuvant-osimertinib regimen, **I would pause the adjuvant-osimertinib regimen 48 hours before & after the ablative therapy** | **I.** If a patient experiences an ex-CNS or CNS recurrence amenable to local consolidative therapy during the adjuvant-osimertinib regimen, **in absence of existing evidence**, I would use the adjuvant-osimertinib regimen in parallel with local consolidative therapy, **considering the risk of toxicities, site of recurrence, method of local consolidative therapy and after discussion with the MDT** |
|  | **B.** If a patient experiences an ex-CNS or CNS recurrence amenable to ablative therapy after completion of the adjuvant-osimertinib regimen, **I would use the ablative therapy prior to rechallenging with osimertinib, irrespective of when the recurrence occurs after the completion of the therapy** | **II.** If a patient experiences an ex-CNS or CNS recurrence amenable to local consolidative therapy after completion of the adjuvant-osimertinib regimen, **I would consider rechallenging with osimertinib as an option after local consolidative therapy, after discussion with MDT** |
| **ex-CNS and/or CNS recurrence during adjuvant-osimertinib regimen** (not amenable to local consolidative therapy) | **C.** If a patient experiences an ex-CNS and/or CNS recurrence during the adjuvant-osimertinib regimen which is not amenable to ablative therapy, **I would continue with 80mg monotherapy osimertinib,** if fits with the best clinical practice | **III.** If a patient experiences **CNS-only** recurrence **not amenable to local consolidative therapy** during the adjuvant-osimertinib regimen, **I would consider continuing with osimertinib as a part of the overall treatment strategy when a patient has asymptomatic progression,** if fits with the best clinical practice |
|  |  | **IV.** In the absence of randomized evidence, if a patient experiences **ex-CNS recurrence** not amenable to local consolidative therapy during the adjuvant-osimertinib regimen, **I would consider continuation with osimertinib as a part of the overall treatment strategy if the patient has asymptomatic progression** and if fits with the current guideline recommendations |
| **ex-CNS and/or CNS recurrence post-adjuvant-osimertinib regimen** (not amenable to local consolidative therapy) | **D.** If a patient experiences an ex-CNS and/or CNS recurrence after completion of the adjuvant-osimertinib regimen, and ablative therapy is not an option**, I would rechallenge with 80mg monotherapy osimertinib,** if fits with the best clinical practice, irrespective of when the recurrence happens after treatment completion | **V.** If a patient experiences an ex-CNS and/or CNS recurrence after completion of the adjuvant-osimertinib regimen, and local consolidative therapy is not an option, **I would rechallenge with monotherapy osimertinib or osimertinib as a part of the overall treatment strategy** irrespective of when the recurrence happens after treatment completion, if fits with the best clinical practice and is **supported by re-biopsy in all feasible cases** |
| **ex-CNS and/or CNS recurrence** (all scenarios) | **E.** If a patient experiences an ex-CNS and/or CNS recurrence during the adjuvant-osimertinib regimen and I choose to continue with osimertinib as part of the overall treatment strategy, **I would continue the osimertinib therapy for at least three years after recurrence or up until a negative CT / MRI result is obtained** | **VI.** If a patient experiences an ex-CNS and/or CNS recurrence during or after the completion of the adjuvant-osimertinib regimen and I choose to continue with osimertinib as part of the overall treatment strategy, **I would continue the osimertinib therapy until the clinical situation mandates a change or stop in therapy** |
|  | **F.** If a patient experiences an ex-CNS and/or CNS after completion of the adjuvant-osimertinib regimen and I choose to rechallenge with osimertinib as part of the overall treatment strategy, **I would rechallenge with osimertinib for at least three years after recurrence or up until a negative CT / MRI result is obtained** |  |
