# Supplementary material for: Treatment decision for recurrences in non-small cell lung cancer during or after adjuvant osimertinib: an international Delphi consensus report
Source: Front Oncol. 2024 Jan 23;13:1330468. doi: 10.3389/fonc.2023.1330468 (PMC10845045; doi:10.3389/fonc.2023.1330468)
Supplement: Supplementary file 2 [file DataSheet_2.docx]

# Survey 1

**A modified-Delphi panel advisory board to evaluate treatment options for patients with EGFRm NSCLC recurrence during or post adjuvant-osimertinib treatment regimen**

First round survey

| Prepared By: |
| --- |
|  |
| CRA International  Date of Preparation: June 2022  Document ID: Z4-46177 |
|  |
|  |

### Patient case studies

| **Case study 1:** A 50-year-old non-Asian male patient with Exon 19 deletion EGFRm NSCLC (stage IIA) underwent a resection surgery and is currently on treatment with adjuvant osimertinib. 6 months after the initiation of the treatment with adjuvant osimertinib, the patient presents with distant recurrence (oligo-metastasis) of Exon 19 deletion EGFRm NSCLC. The patient has been previously treated with adjuvant chemotherapy and has a performance status (PS) score ≤ 1. | |
| --- | --- |
| 1. Considering the patient history, the treatment with adjuvant osimertinib after resection surgery is appropriate | |
| Agreement rating on a 9-point **Likert scale** |  |
| *Rationale/Comments* |  |
| 1. During the treatment regimen with adjuvant osimertinib, I would monitor this patient with… | |
| 1. [Placeholder for the method 1 and frequency] |  |
| 1. [Placeholder for the method 2 and frequency] |  |
| 1. [Placeholder for the method 3 and frequency] |  |
| *Rationale/Comments* |  |
| 1. Upon suspicion of tumour recurrence, I will perform the following diagnostic work-up to confirm the recurrence type *(please mention all the techniques used to get a confirmed diagnosis)* | |
| 1. Imaging tests (CT, PET and/or MRI scan) |  |
| 1. Tissue biomarker testing |  |
| 1. Liquid biopsy |  |
| 1. [Placeholder for additional methodology] |  |
| *Rationale/Comments* |  |
| 1. How does your approach for diagnostic work-up change if the patient presented with **disseminated distant recurrence**? | |
| 1. No change, the diagnostic approach will be same as that for oligometastatic distant recurrence |  |
| 1. [Placeholder for the diagnostic work-up 1] |  |
| 1. [Placeholder for the diagnostic work-up 2] |  |
| *Rationale/Comments* |  |
| 1. Upon confirmation of the **oligometastatic distant recurrence**, the treatment approach for the patient will be following… | |
| 1. [Placeholder for the treatment approach 1] |  |
| 1. [Placeholder for the treatment approach 2] |  |
| *Rationale/Comments (please indicate the major influencing factors?)* |  |
| 1. How does your treatment approach change if the patient presented with **disseminated distant recurrence**? | |
| 1. No change, the treatment approach will be same as that for oligometastatic distant recurrence |  |
| 1. [Placeholder for the treatment approach 1] |  |
| 1. [Placeholder for the treatment approach 2] |  |
| *Rationale/Comments* |  |
| 1. How does your treatment approach change if the oligometastatic or disseminated distant **recurrence occurs during the adjuvant-osimertinib regimen at a time point 1.5 years from the start of the regimen?** | |
| 1. No change |  |
| 1. [Placeholder for the treatment approach 1 for oligometastatic distant tumour] |  |
| 1. [Placeholder for the treatment approach 2 for oligometastatic distant tumour] |  |
| *Rationale/Comments* |  |
| 1. No change |  |
| 1. [Placeholder for the treatment approach 1 for disseminated distant tumour] |  |
| 1. [Placeholder for the treatment approach 2 for disseminated distant tumour] |  |
| *Rationale/Comments* |  |

| **Case study 2:** A 75-year-old non-Asian female patient with Exon 21 L858R EGFRm NSCLC (stage IIA) underwent a resection surgery and was subsequently treated with adjuvant osimertinib for 3 years. 6 months after the completion of adjuvant osimertinib regimen, the patient presents with distant recurrence (oligo-metastasis) of Exon 21 L858R EGFRm NSCLC. The patient has been previously treated with adjuvant chemotherapy and has a performance status (PS) score ≤ 1. | |
| --- | --- |
| 1. Considering the patient history, the treatment with adjuvant osimertinib after resection surgery is appropriate | |
| Agreement rating on a 9-point **Likert scale** |  |
| *Rationale/Comments* |  |
| 1. During the treatment regimen with adjuvant osimertinib, I would monitor my patients through… | |
| 1. [Placeholder for the method 1 and frequency] |  |
| 1. [Placeholder for the method 2 and frequency] |  |
| *Rationale/Comments* |  |
| 1. After the treatment regimen with adjuvant osimertinib, I would monitor my patients through… | |
| 1. No change, the monitoring approach will be same as before the completion of adjuvant osimertinib regimen |  |
| 1. [Placeholder for the method 1 and frequency] |  |
| 1. [Placeholder for the method 2 and frequency] |  |
| *Rationale/Comments* |  |
| 1. Upon suspicion of tumour recurrence, I will perform the following diagnostic work-up to confirm the recurrence type *(please mention all the techniques used to get a confirmed diagnosis)* | |
| 1. Imaging tests (CT, PET and/or MRI scan) |  |
| 1. Tissue biomarker testing |  |
| 1. Liquid biopsy |  |
| 1. [Placeholder for additional methodology] |  |
| *Rationale/Comments* |  |
| 1. How does your approach for diagnostic work-up change if the patient presented with disseminated distant recurrence? | |
| 1. No change, the diagnostic approach will be the same as that for oligometastatic distant recurrence |  |
| 1. [Placeholder for the diagnostic work-up 1] |  |
| 1. [Placeholder for the diagnostic work-up 2] |  |
| *Rationale/Comments* |  |
| 1. Upon confirmation of the oligometastatic distant recurrence, the treatment approach for the patient will be following… | |
| 1. [Placeholder for the treatment approach 1] |  |
| 1. [Placeholder for the treatment approach 2] |  |
| *Rationale/Comments (please indicate the major influencing factors?)* |  |
| 1. How does your treatment approach change if the patient presented with **disseminated distant recurrence?** | |
| 1. No change, the treatment approach will be the same as that for oligometastatic distant recurrence |  |
| 1. [Placeholder for the treatment approach 1] |  |
| 1. [Placeholder for the treatment approach 2] |  |
| *Rationale/Comments* |  |
| 1. How does your treatment approach change if the oligometastatic or disseminated distant **recurrence occurred 3 months after the completion of the adjuvant osimertinib regimen**? | |
| 1. No change |  |
| 1. [Placeholder for the treatment approach 1 for oligometastatic distant tumour] |  |
| 1. [Placeholder for the treatment approach 2 for oligometastatic distant tumour] |  |
| *Rationale/Comments* |  |
| 1. No change |  |
| 1. [Placeholder for the treatment approach 1 for disseminated distant tumour] |  |
| 1. [Placeholder for the treatment approach 2 for disseminated distant tumour] |  |
| *Rationale/Comments* |  |
| 1. How does your treatment approach change if the oligometastatic or disseminated distant **recurrence occurred 1 year after the completion of the adjuvant osimertinib regimen**? | |
| 1. No change |  |
| 1. [Placeholder for the treatment approach 1 for oligometastatic distant tumour] |  |
| 1. [Placeholder for the treatment approach 2 for oligometastatic distant tumour] |  |
| *Rationale/Comments* |  |
| 1. No change |  |
| 1. [Placeholder for the treatment approach 1 for disseminated distant tumour] |  |
| 1. [Placeholder for the treatment approach 2 for disseminated distant tumour] |  |
| *Rationale/Comments* |  |

| **Case study 3:** A 65-year-old Asian male patient with Exon 19 deletion EGFRm NSCLC (stage IIA) underwent a resection surgery and is currently on treatment with adjuvant osimertinib. 1.5 years after the initiation of the treatment with adjuvant osimertinib, the patient presents with local recurrence of Exon 19 deletion EGFRm NSCLC. The patient has not been previously treated with adjuvant chemotherapy and has a performance status (PS) score ≤ 1. | |
| --- | --- |
| 1. Considering the patient history, the treatment with adjuvant osimertinib after resection surgery is appropriate | |
| Agreement rating on a 9-point **Likert scale** |  |
| *Rationale/Comments* |  |
| 1. During the treatment regimen with adjuvant osimertinib, I would monitor my patients through… | |
| 1. [Placeholder for the method 1 and frequency] |  |
| 1. [Placeholder for the method 2 and frequency] |  |
| *Rationale/Comments* |  |
| 1. Upon suspicion of tumour recurrence, I will perform the following diagnostic work-up to confirm the recurrence type *(please mention all the techniques used to get a confirmed diagnosis)* | |
| 1. Imaging tests (CT, PET and/or MRI scan) |  |
| 1. Tissue biomarker testing |  |
| 1. Liquid biopsy |  |
| 1. [Placeholder for additional methodology] |  |
| *Rationale/Comments* |  |
| 1. Upon confirmation of the local recurrence, the treatment approach for the patient will be following… | |
| 1. [Placeholder for the treatment approach 1] |  |
| 1. [Placeholder for the treatment approach 2] |  |
| *Rationale/Comments (please indicate the major influencing factors?)* |  |
| 1. How does your treatment approach differ for the patient who was initially diagnosed with **Exon 21 L858R EGFRm NSCLC** and later presents with local recurrence of the same mutation type? Please consider other patient characteristics to remain unchanged. | |
| 1. No change, the treatment approach will be same as that for a patient with local recurrence of Exon 19 deletion EGFRm NSCLC |  |
| 1. [Placeholder for the treatment approach 1] |  |
| 1. [Placeholder for the treatment approach 2] |  |
| *Rationale/Comments (please indicate the major influencing factors?)* |  |
| 1. How does your treatment approach differ if local recurrence occurred **after 3 months, 6 months or 1 year of the completion of the adjuvant osimertinib regimen**? | |
| 1. No change for recurrence after 3 months |  |
| 1. [Placeholder for the treatment approach 1 for recurrence after 3 months] |  |
| 1. [Placeholder for the treatment approach 2 for recurrence after 3 months] |  |
| *Rationale/Comments (please indicate the major influencing factors?)* |  |
| 1. No change for recurrence after 6 months |  |
| 1. [Placeholder for the treatment approach 1 for recurrence after 6 months] |  |
| 1. [Placeholder for the treatment approach 2 for recurrence after 6 months] |  |
| *Rationale/Comments (please indicate the major influencing factors?)* |  |
| 1. No change for recurrence after 1 year |  |
| 1. [Placeholder for the treatment approach 1 for recurrence after 1 year] |  |
| 1. [Placeholder for the treatment approach 2 for recurrence after 1 year] |  |
| *Rationale/Comments (please indicate the major influencing factors?)* |  |
| 1. Would your responses to the previous question (Question 22) be the same if the patient was initially diagnosed with **Exon 21 L858R EGFRm NSCLC** and later presents with local recurrence of the same mutation type after 3 months, 6 months or 1 year of the completion of the adjuvant osimertinib regimen? | |
| 1. Yes (for all three distinct recurrence timepoints) |  |
| 1. No |  |
| *Rationale/Comments (in case of option ‘No’, please mention the recurrence timepoint(s) and rationale behind different treatment approaches)* |  |

| **Case study 4:** A 75-year-old non-Asian male patient with Exon 19 deletion EGFRm NSCLC (stage IIA) underwent a resection surgery and is currently on treatment with adjuvant osimertinib. 6 months after the initiation of the treatment with adjuvant osimertinib, the patient presents with oligometastatic recurrence of Exon 19 deletion EGFRm NSCLC in the CNS. The patient has been previously treated with adjuvant chemotherapy and has a performance status (PS) score ≤ 1. | |
| --- | --- |
| 1. Considering the patient history, the treatment with adjuvant osimertinib after resection surgery is appropriate | |
| Agreement rating on a 9-point **Likert scale** |  |
| *Rationale/Comments* |  |
| 1. Upon suspicion of tumour recurrence, I will perform the following diagnostic work-up to confirm the recurrence type *(please mention all the techniques used to get a confirmed diagnosis)* | |
| 1. Imaging tests (CT, PET and/or MRI scan) |  |
| 1. Tissue biomarker testing |  |
| 1. Liquid biopsy |  |
| 1. [Placeholder for additional methodology] |  |
| *Rationale/Comments* |  |
| 1. Upon confirmation of the oligometastatic CNS recurrence, the treatment approach for the patient will be following… | |
| 1. [Placeholder for the treatment approach 1] |  |
| 1. [Placeholder for the treatment approach 2] |  |
| *Rationale/Comments (please indicate the major influencing factors?)* |  |
| 1. How does your treatment approach differ for the patient who was initially diagnosed with **Exon 21 L858R EGFRm NSCLC** and later presents with oligometastatic CNS recurrence of Exon 21 L858R EGFRm NSCLC? Please consider other patient characteristics to remain unchanged. | |
| 1. No change, the treatment approach will be the same as that for a patient with Exon 19 deletion EGFRm NSCLC oligometastatic CNS recurrence |  |
| 1. [Placeholder for the treatment approach 1] |  |
| 1. [Placeholder for the treatment approach 2] |  |
| *Rationale/Comments (please indicate the major influencing factors?)* |  |
| 1. How does your treatment approach differ for the patient with **disseminated CNS recurrence of Exon 19 deletion EGFRm NSCLC**? | |
| 1. No change |  |
| 1. [Placeholder for the treatment approach 1 for disseminated CNS recurrence] |  |
| 1. [Placeholder for the treatment approach 2 for disseminated CNS recurrence] |  |
| *Rationale/Comments (please indicate the major influencing factors?)* |  |

| **Case study 5:** A 50-year-old non-Asian female patient with Exon 21 L858R EGFRm NSCLC (stage IIA) underwent a resection surgery and was subsequently treated with adjuvant osimertinib for 3 years. 6 months after the completion of adjuvant osimertinib regimen, the patient presents with oligo-metastasis CNS recurrence of Exon 21 L858R EGFRm NSCLC. The patient has been previously treated with adjuvant chemotherapy and has a performance status (PS) score ≤ 1. | |
| --- | --- |
| 1. Considering the patient history, the treatment with adjuvant osimertinib after resection surgery is appropriate | |
| Agreement rating on a 9-point **Likert scale** |  |
| *Rationale/Comments* |  |
| 1. Upon confirmation of the oligometastatic CNS recurrence, the treatment approach for the patient will be the following… | |
| 1. [Placeholder for the treatment approach 1] |  |
| 1. [Placeholder for the treatment approach 2] |  |
| *Rationale/Comments (please indicate the major influencing factors?)* |  |
| 1. How does your treatment approach differ if the patient presented with **disseminated CNS recurrence**? | |
| 1. No change, the treatment approach will be same as that for oligometastatic CNS recurrence |  |
| 1. [Placeholder for the treatment approach 1] |  |
| 1. [Placeholder for the treatment approach 2] |  |
| *Rationale/Comments (please indicate the major influencing factors?)* |  |
| 1. How does your treatment approach differ if oligometastatic or disseminated CNS **recurrence occurred 3 months after the completion of the adjuvant osimertinib regimen**? | |
| 1. No change |  |
| 1. [Placeholder for the treatment approach 1 for oligometastatic CNS recurrence] |  |
| 1. [Placeholder for the treatment approach 2 for oligometastatic CNS recurrence] |  |
| *Rationale/Comments (please indicate the major influencing factors?)* |  |
| 1. No change |  |
| 1. [Placeholder for the treatment approach 1 for disseminated CNS recurrence] |  |
| 1. [Placeholder for the treatment approach 2 for disseminated CNS recurrence] |  |
| *Rationale/Comments (please indicate the major influencing factors?)* |  |
| 1. How does your treatment approach differ if oligometastatic or disseminated CNS **recurrence occurred 1 year after the completion of the adjuvant osimertinib regimen**? | |
| 1. No change |  |
| 1. [Placeholder for the treatment approach 1 for oligometastatic CNS recurrence] |  |
| 1. [Placeholder for the treatment approach 2 for oligometastatic CNS recurrence] |  |
| *Rationale/Comments (please indicate the major influencing factors?)* |  |
| 1. No change |  |
| 1. [Placeholder for the treatment approach 1 for disseminated CNS recurrence] |  |
| 1. [Placeholder for the treatment approach 2 for disseminated CNS recurrence] |  |
| *Rationale/Comments (please indicate the major influencing factors?)* |  |

| **Case study 6:** A 65-year-old Asian female patient with Exon 21 L858R EGFRm NSCLC (stage IIA) underwent a resection surgery and is currently on treatment with adjuvant osimertinib. 1.5 years after the initiation of the treatment with adjuvant osimertinib, the patient presents with distant and CNS recurrence of Exon 21 L858R EGFRm NSCLC. The patient has not been previously treated with adjuvant chemotherapy and has a performance status (PS) score ≤ 1. | |
| --- | --- |
| 1. Considering the patient history, the treatment with adjuvant osimertinib after resection surgery is appropriate | |
| Agreement rating on a 9-point **Likert scale** |  |
| *Rationale/Comments* |  |
| 1. Upon suspicion of tumour recurrence, I will perform the following diagnostic work-up to confirm the recurrence type *(please mention all the techniques used to get a confirmed diagnosis)* | |
| 1. Imaging tests (CT, PET and/or MRI scan) |  |
| 1. Tissue biomarker testing |  |
| 1. Liquid biopsy |  |
| 1. [Placeholder for additional methodology] |  |
| *Rationale/Comments* |  |
| 1. Is the diagnostic work-up always able to accurately detect both distant and CNS recurrence? | |
| 1. Yes |  |
| 1. No |  |
| *Rationale/Comments (in case of option ‘No’)* |  |
| 1. Upon confirmation of the recurrence, the treatment approach for the patient will be following… | |
| 1. [Placeholder for the treatment approach 1] |  |
| 1. [Placeholder for the treatment approach 2] |  |
| *Rationale/Comments (please indicate the major influencing factors?)* |  |
| 1. How does your treatment approach differ if the **recurrence occurred 3 months after the completion of the adjuvant osimertinib regimen**? | |
| 1. No change |  |
| 1. [Placeholder for the treatment approach 1] |  |
| 1. [Placeholder for the treatment approach 2] |  |
| *Rationale/Comments (please indicate the major influencing factors?)* |  |
| 1. How does your treatment approach differ if the **recurrence occurred 6 months after the completion of the adjuvant osimertinib regimen**? | |
| 1. No change |  |
| 1. [Placeholder for the treatment approach 1] |  |
| 1. [Placeholder for the treatment approach 2] |  |
| *Rationale/Comments (please indicate the major influencing factors?)* |  |
| 1. How does your treatment approach differ if the **recurrence occurred 1 year after the completion of the adjuvant osimertinib regimen**? | |
| 1. No change |  |
| 1. [Placeholder for the treatment approach 1] |  |
| 1. [Placeholder for the treatment approach 2] |  |
| *Rationale/Comments (please indicate the major influencing factors?)* |  |

# Survey 2

**A modified-Delphi panel advisory board to evaluate treatment options for patients with EGFRm NSCLC recurrence during or post adjuvant-osimertinib treatment regimen**

Second round survey

| Prepared By: |
| --- |
|  |
| CRA International  Date of Preparation: Aug 2022  Document ID: Z4-47826 |
|  |

**Patient monitoring & diagnostic workup**

Please note that all responses should consider tumour recurrence in an EGFR-mutated NSCLC patient currently being treated with adjuvant-osimertinib or who has recently completed the adjuvant-osimertinib regimen

| 1. In addition to monitoring patients in adjuvant-osimertinib through CT/PET CT scan and Brain MRI, I would conduct: | |
| --- | --- |
| **Options** | **Your response** |
| 1. Minimal residual disease (MRD) test   *Rating on the 1-9 Likert scale* |  |
| 1. Blood tests, e.g., CEA (carcinoembryonic antigen) assay   *Rating on the 1-9 Likert scale* |  |
| 1. Others   *Rating on the 1-9 Likert scale* |  |
| *Please provide the name (in case of the option ‘Others’), frequency and rationale for the techniques used* |  |
|  | |
| 1. During the diagnostic work-up, I perform liquid biopsies when: | |
| 1. Tissue biopsy is difficult   *Rating on the 1-9 Likert scale* |  |
| 1. Non-invasive procedure is preferred by the patient   *Rating on the 1-9 Likert scale* |  |
| 1. Non-invasive procedure is preferred by the doctor   *Rating on the 1-9 Likert scale* |  |
| 1. I do not perform liquid biopsies   *Please mark with X* |  |
| 1. Others   *Rating on the 1-9 Likert scale* |  |
| *Please provide an example of a situation where you would consider liquid biopsy* |  |
|  | |
| 1. In which clinical situation of NSCLC progression would you perform next-generation sequencing (NGS) or other molecular analysis tools for determining the molecular characteristics of the tumor sample? *Please mention ‘Always’ in case molecular characteristic is always determined* | |
| 1. *[Please replace this placeholder with your response]* | |
| 1. *[Please replace this placeholder with your alternate response 1]* | |
| 1. *[Please replace this placeholder with your alternate response 2]* | |
| *Your rationale/comments* |  |
|  | |
| 1. I would continue the prescribed osimertinib regimen during the entire diagnostic work-up for confirming the recurrence/type of recurrence | |
| *Rating on the 1-9 Likert scale* |  |
| *Your rationale/comments* |  |
|  | |
| 1. Upon confirmation of the recurrence/type of recurrence, I would continue the prescribed osimertinib regimen until a treatment option can be implemented | |
| *Rating on the 1-9 Likert scale* |  |
| *Your rationale/comments* |  |

### Treatment approach for ex-CNS recurrence during or after adjuvant osimertinib for the treatment of stage IB-IIIA EGFRm NSCLC

| 1. If 6 months after the start of the adjuvant-osimertinib regimen, a distant recurrence presented ex-CNS and lesions were deemed amenable for ablative therapy by the multidisciplinary team (MDT), I would | | | |
| --- | --- | --- | --- |
| **First treatment approach** | Use ablative therapy (including surgery, radio therapy, radiofrequency or others) that is most suitable for the patient | Use systemic chemotherapy or other systemic non-TKI options (e.g., immune-oncology therapy) either alone or in combination  ***[Insert therapy of choice]*** | ***[Insert alternate treatment approach]*** |
| *Rating on 1-9 Likert scale* |  |  |  |
| *Select one*   1. *Followed by* 2. *In parallel to* |  |  |  |
| *[Insert your treatment of choice]* |  |  |  |
| I would continue treatment with adjuvant-osimertinib  *Rating on the 1-9 Likert scale* |  |  |  |
| **In case the treatment with adjuvant osimertinib is continued**, please provide details if its use is impacted in any way (e.g., temporary pause when ablative therapy is ongoing, change in frequency or dosing) |  |  |  |
| *Your rationale/ comment* |  |  |  |

| 1. I would use the same therapy options I mentioned in response to question 6, if the same described recurrence occurred **3 months after the start** of the adjuvant-osimertinib regimen | |
| --- | --- |
| *Rating on the 1-9 Likert scale* |  |
| *Please provide an example and rationale for when your approach would be different (if the case)* |  |
| I would continue the treatment with adjuvant osimertinib  *Rating on the 1-9 Likert scale* |  |
| *Your rationale/ comment for response to continuation of adjuvant osimertinib regimen - please comment on if you would change the regimen in any way* |  |
|  | |
| 1. I would use the same therapy options I mentioned in response to question 6, if the same described recurrence occurred **1.5 years after the start** of the adjuvant-osimertinib regimen | |
| *Rating on the 1-9 Likert scale* |  |
| *Please provide an example and rationale for when your approach would be different (if the case)* |  |
| I would continue the treatment with adjuvant osimertinib  *Rating on the 1-9 Likert scale* |  |
| *Your rationale/ comment for response to continuation of adjuvant osimertinib regimen - please comment on if you would change the regimen in any way* |  |
|  | |
| 1. I would **change the therapy approach** mentioned in response to question 6, if the same described recurrence occurred **3 months after the completion** of the adjuvant-osimertinib regimen | |
| *Rating on the 1-9 Likert scale* |  |
| *In case of rating ≥ 3, please describe your alternate treatment approach below:*  *[Please replace this placeholder with your alternate treatment strategy]* | |
| I would rechallenge with osimertinib in this scenario  *Rating on the 1-9 Likert scale* |  |
| *Your rationale/ comment for response to rechallenge with osimertinib regimen* |  |
|  | |
| 1. I would **change the therapy approach** mentioned in response to question 6, if the same described recurrence occurred **1 year after the completion** of the adjuvant-osimertinib regimen | |
| *Rating on the 1-9 Likert scale* |  |
| *In case of rating ≥ 3, please describe your alternate treatment approach below:*  *[Please replace this placeholder with your alternate treatment strategy]* | |
| I would rechallenge with osimertinib in this scenario  *Rating on the 1-9 Likert scale* |  |
| *Your rationale/ comment for response to rechallenge with osimertinib regimen* |  |
|  | |
| 1. I would **change the therapy approach** mentioned in response to question 6, if the same described recurrence occurred **3 years after the completion** of the adjuvant-osimertinib regimen | |
| *Rating on the 1-9 Likert scale* |  |
| *In case of rating ≥ 3, please describe your alternate treatment approach below:*  *[Please replace this placeholder with your alternate treatment strategy]* | |
| I would rechallenge with osimertinib in this scenario  *Rating on the 1-9 Likert scale* |  |
| *Your rationale/ comment for response to rechallenge with osimertinib regimen* |  |

| 1. 6 months after the start of the adjuvant-osimertinib regimen, a distant recurrence presents ex-CNS which is deemed not amenable for ablative therapy by the multidisciplinary team (MDT). Instead, the MDT agree to treat the patient with a systemic chemotherapy considering the re-biopsy results show no targetable resistance mutation | |
| --- | --- |
| I agree with the MDTs approach  *Rating on the 1-9 Likert scale* |  |
| In case you do not agree with the overall treatment approach (rating ≤ 6), please provide details about the alternate suitable treatment approach |  |
| I would continue treatment with adjuvant-osimertinib in parallel to the treatment approach  *Rating on the 1-9 Likert scale* |  |
| I would continue treatment with adjuvant-osimertinib following the treatment approach  *Rating on the 1-9 Likert scale* |  |
| **In case the treatment with adjuvant osimertinib is continued**, please provide details on how it would be used in parallel and/or following treatment (e.g., is there a change in frequency, dosing, pause time) |  |
| *Your rationale/ comment for the above* |  |

| 1. I would use adjuvant-osimertinib the same way I described in questions 12, if the same described recurrence occurred **3 months from the start** of the adjuvant-osimertinib regimen | |
| --- | --- |
| *Rating on the 1-9 Likert scale* |  |
| *Please provide an example and rationale for when your approach would be different (if the case)* |  |
|  | |
| 1. I would use adjuvant-osimertinib the same way I described in questions 12, if the same described recurrence occurred **1.5 years from the start** of the adjuvant-osimertinib regimen | |
| *Rating on the 1-9 Likert scale* |  |
| *Please provide an example and rationale for when your approach would be different (if the case)* |  |
|  | |
| 1. I would **change the therapy approach** mentioned in response to question 12, if the same described recurrence occurred **3 months after the completion** of the adjuvant-osimertinib regimen | |
| *Rating on the 1-9 Likert scale* |  |
| *In case of rating ≥ 3, please describe your alternate treatment approach below:*  *[Please replace this placeholder with your alternate treatment strategy]* | |
| I would rechallenge with osimertinib in this scenario  *Rating on the 1-9 Likert scale* |  |
| **In case of rechallenge with osimertinib**, please provide details on how it would be used in parallel and/or following treatment (e.g., is there a change in frequency, dosing, pause time) |  |
|  | |
| 1. I would **change the therapy approach** mentioned in response to question 12, if the same described recurrence occurred **1 year after the completion** of the adjuvant-osimertinib regimen | |
| *Rating on the 1-9 Likert scale* |  |
| *In case of rating ≥ 3, please describe your alternate treatment approach below:*  *[Please replace this placeholder with your alternate treatment strategy]* | |
| I would rechallenge with osimertinib in this scenario  *Rating on the 1-9 Likert scale* |  |
| **In case of rechallenge with osimertinib**, please provide details on how it would be used in parallel and/or following treatment (e.g., is there a change in frequency, dosing, pause time) |  |
|  | |
| 1. I would **change the therapy approach** mentioned in response to question 12, if the same described recurrence occurred **3 years after the completion** of the adjuvant-osimertinib regimen | |
| *Rating on the 1-9 Likert scale* |  |
| *In case of rating ≥ 3, please describe your alternate treatment approach below:*  *[Please replace this placeholder with your alternate treatment strategy]* | |
| I would rechallenge with osimertinib in this scenario  *Rating on the 1-9 Likert scale* |  |
| **In case of rechallenge with osimertinib**, please provide details on how it would be used in parallel and/or following treatment (e.g., is there a change in frequency, dosing, pause time) |  |

### Treatment approach for CNS recurrence (brain metastases) during or after adjuvant osimertinib for the treatment of stage IB-IIIA EGFRm NSCLC

**Note:** For the following questions please pay attention to the changes in recurrence scenario that have been **highlighted in orange**

| 1. If 6 months after the start of the adjuvant-osimertinib regimen, a distant non-symptomatic recurrence presented in the brain (PS = 1) and lesions were deemed amenable for ablative therapy by the multidisciplinary team (MDT), I would | | | |
| --- | --- | --- | --- |
| First treatment approach | Use ablative therapy (including surgery, radio therapy, radiofrequency or others) that is most suitable for the patient | Use systemic chemotherapy or other systemic non-TKI options (e.g., immune-oncology therapy) either alone or in combination  ***[Insert therapy of choice]*** | ***[Insert alternate treatment approach]*** |
| *Rating on 1-9 Likert scale* |  |  |  |
| *Select one*   1. *Followed by* 2. *In parallel to* |  |  |  |
| *[Insert your treatment of choice]* |  |  |  |
| I would continue treatment with adjuvant-osimertinib  *Rating on the 1-9 Likert scale* |  |  |  |
| **In case the treatment with adjuvant osimertinib is continued**, please provide details if its use is impacted in any way (e.g., temporary pause when ablative therapy is ongoing, change in frequency or dosing) |  |  |  |
| *Your rationale/ comment* |  |  |  |

| 1. I would use the same therapy options I mentioned in response to question 18, if the same described recurrence occurred **3 months after the start** of the adjuvant-osimertinib regimen | |
| --- | --- |
| *Rating on the 1-9 Likert scale* |  |
| *Please provide an example and rationale for when your approach would be different (if the case)* |  |
| I would continue the treatment with adjuvant osimertinib  *Rating on the 1-9 Likert scale* |  |
| *Your rationale/ comment for response to continuation of adjuvant osimertinib regimen - please comment on if you would change the regimen in any way* |  |
|  | |
| 1. I would use the same therapy options I mentioned in response to question 18, if the same described recurrence occurred **1.5 years after the start** of the adjuvant-osimertinib regimen | |
| *Rating on the 1-9 Likert scale* |  |
| *Please provide an example and rationale for when your approach would be different (if the case)* |  |
| I would continue the treatment with adjuvant osimertinib  *Rating on the 1-9 Likert scale* |  |
| *Your rationale/ comment for response to continuation of adjuvant osimertinib regimen - please comment on if you would change the regimen in any way* |  |
|  | |
| 1. I would **change the therapy approach** mentioned in response to question 18, if the same described recurrence occurred **3 months after the completion** of the adjuvant-osimertinib regimen | |
| *Rating on the 1-9 Likert scale* |  |
| *In case of rating ≥ 3, please describe your alternate treatment approach below:*  *[Please replace this placeholder with your alternate treatment strategy]* | |
| I would rechallenge with osimertinib in this scenario  *Rating on the 1-9 Likert scale* |  |
| **In case of rechallenge with osimertinib**, please provide details on how it would be used in parallel and/or following treatment (e.g., is there a change in frequency, dosing, pause time) |  |
|  | |
| 1. I would **change the therapy approach** mentioned in response to question 18, if the same described recurrence occurred **1 year after the completion** of the adjuvant-osimertinib regimen | |
| *Rating on the 1-9 Likert scale* |  |
| *In case of rating ≥ 3, please describe your alternate treatment approach below:*  *[Please replace this placeholder with your alternate treatment strategy]* | |
| I would rechallenge with osimertinib in this scenario  *Rating on the 1-9 Likert scale* |  |
| **In case of rechallenge with osimertinib**, please provide details on how it would be used in parallel and/or following treatment (e.g., is there a change in frequency, dosing, pause time) |  |
|  | |
| 1. I would **change the therapy approach** mentioned in response to question 18, if the same described recurrence occurred **3 years after the completion** of the adjuvant-osimertinib regimen | |
| *Rating on the 1-9 Likert scale* |  |
| *In case of rating ≥ 3, please describe your alternate treatment approach below:*  *[Please replace this placeholder with your alternate treatment strategy]* | |
| I would rechallenge with osimertinib in this scenario  *Rating on the 1-9 Likert scale* |  |
| **In case of rechallenge with osimertinib**, please provide details on how it would be used in parallel and/or following treatment (e.g., is there a change in frequency, dosing, pause time) |  |
|  | |

| 1. I would **change the therapy approach** mentioned in response to question 18, if a **distant symptomatic recurrence presented in the brain (PS = 1)** and **lesions were deemed amenable for ablative therapy** by the multidisciplinary team (MDT) | |
| --- | --- |
| *Rating on the 1-9 Likert scale* |  |
| *In case of rating ≥ 3, please describe your alternate treatment approach below:*  *[Please replace this placeholder with your alternate treatment strategy]* | |
| I would continue the treatment with adjuvant osimertinib  *Rating on the 1-9 Likert scale* |  |
| *Your rationale/ comment for response to continuation of adjuvant osimertinib regimen - please comment on if you would change the regimen in any way* |  |
| I would use the same approach as above regardless of when this type of recurrence occurred (i.e., any time during or after completion with adjuvant-osimertinib therapy).  *Rating on the 1-9 Likert scale* |  |
| *Your rationale/ comment to the above response and how osimertinib would be used (if at all)* |  |
|  | |
| 1. I would **change the therapy approach** mentioned in response to question 18, if a **distant non-symptomatic recurrence presented in the brain (PS = 1)** and **lesions were deemed not amenable for ablative therapy** by the multidisciplinary team (MDT) | |
| *Rating on the 1-9 Likert scale* |  |
| *In case of rating ≥ 3, please describe your alternate treatment approach below:*  *[Please replace this placeholder with your alternate treatment strategy]* | |
| I would continue the treatment with adjuvant osimertinib  *Rating on the 1-9 Likert scale* |  |
| *Your rationale/ comment for response to continuation of adjuvant osimertinib regimen - please comment on if you would change the regimen in any way* |  |
| I would use the same approach as above regardless of when this type of recurrence occurred (i.e., any time during or after completion with adjuvant-osimertinib therapy).  *Rating on the 1-9 Likert scale* |  |
| *Your rationale/ comment to the above response and how osimertinib would be used (if at all)* |  |
|  | |
| 1. I would **change the therapy approach** mentioned in response to question 18, if a **distant symptomatic recurrence presented in the brain** **(PS = 1)** and **lesions were deemed not amenable for ablative therapy** by the multidisciplinary team (MDT) | |
| *Rating on the 1-9 Likert scale* |  |
| *In case of rating ≥ 3, please describe your alternate treatment approach below:*  *[Please replace this placeholder with your alternate treatment strategy]* | |
| I would continue the treatment with adjuvant osimertinib  *Rating on the 1-9 Likert scale* |  |
| *Your rationale/ comment for response to continuation of adjuvant osimertinib regimen - please comment on if you would change the regimen in any way* |  |
| I would use the same approach as above regardless of when this type of recurrence occurred (i.e., any time during or after completion with adjuvant-osimertinib therapy).  *Rating on the 1-9 Likert scale* |  |
| *Your rationale/ comment to the above response and how osimertinib would be used (if at all)* |  |
|  | |
| 1. I would **change the therapy approach** mentioned in response to question 18, if a **distant non-symptomatic recurrence presented in both the brain and ex-CNS (PS = 1)** and **lesions in the brain were deemed not amenable for ablative therapy** by the multidisciplinary team (MDT) | |
| *Rating on the 1-9 Likert scale* |  |
| *In case of rating ≥ 3, please describe your alternate treatment approach below:*  *[Please replace this placeholder with your alternate treatment strategy]* | |
| I would continue the treatment with adjuvant osimertinib  *Rating on the 1-9 Likert scale* |  |
| *Your rationale/ comment for response to continuation of adjuvant osimertinib regimen - please comment on if you would change the regimen in any way* |  |
| I would use the same approach as above regardless of when this type of recurrence occurred (i.e., any time during or after completion with adjuvant-osimertinib therapy).  *Rating on the 1-9 Likert scale* |  |
| *Your rationale/ comment to the above response and how osimertinib would be used (if at all)* |  |
|  | |
| 1. I would **change the therapy approach** mentioned in response to question 18, if a **distant symptomatic recurrence presented in both the brain and ex-CNS (PS = 1)** and **lesions in the brain were deemed not amenable for ablative therapy** by the multidisciplinary team (MDT) | |
| *Rating on the 1-9 Likert scale* |  |
| *In case of rating ≥ 3, please describe your alternate treatment approach below:*  *[Please replace this placeholder with your alternate treatment strategy]* | |
| I would continue the treatment with adjuvant osimertinib  *Rating on the 1-9 Likert scale* |  |
| *Your rationale/ comment for response to continuation of adjuvant osimertinib regimen - please comment on if you would change the regimen in any way* |  |
| I would use the same approach as above regardless of when this type of recurrence occurred (i.e., any time during or after completion with adjuvant-osimertinib therapy).  *Rating on the 1-9 Likert scale* |  |
| *Your rationale/ comment to the above response and how osimertinib would be used (if at all)* |  |

### Additional information

| 1. What are your key considerations while deciding a treatment for patients that recur after completion of the adjuvant osimertinib regimen? | |
| --- | --- |
| 1. *[Insert key consideration 1]* |  |
| 1. *[Insert key consideration 2]* |  |
| 1. *[Insert key consideration 3]* |  |
| 1. *[Insert key consideration 4]* |  |
| *Your rationale/comments* |  |
